# Increasing dietary oat fibre decreases the permeability of intestinal mucus

**DOI:** 10.1016/j.jff.2016.08.018

**Published:** 2016-10

**Authors:** Alan Mackie, Neil Rigby, Pascale Harvey, Balazs Bajka

**Affiliations:** a School of Food Science and Nutrition, University of Leeds, Leeds LS29JT, UK; b Institute of Food Research, Norwich Research Park, Colney Lane, Norwich NR4 7UA, UK; c Easton Otley College, Easton, Norwich NR9 5DX, UK

**Keywords:** Dietary fibre, Beta-glucan, Porcine, Mucus, Permeability, Diffusion

## Abstract

This study investigates the influence of the dietary fibre β-glucan on nutrient composition and mucus permeability.

Pigs were fed a standard diet or a diet containing twice the β-glucan content for 3 days (n = 5 per group), followed by the collection of small intestinal mucus and tissue samples. Samples of the consumed diets were subjected to *in vitro* digestion to determine β-glucan release, nutrient profile and assessment of mucus permeability.

*In vitro* digestion of the diets indicated that 90% of the β-glucan was released in the proximal small intestine. Measurements of intestinal mucus showed a reduction in permeability to 100 nm latex beads and also lipid from the digested enhanced β-glucan diet.

The data from this study show for the first time that reducing mass transfer of bile and lipid through the intestinal mucus layer may be one way in which this decrease in bile reabsorption by soluble fibre is enabled.

## Highlights

• Comparison was made between an enhanced β-glucan diet versus a control diet in pigs.
• *In vitro* digestion showed that 90% of the β-glucan was solubilised in the proximal small intestine.
• The permeability of the intestinal mucus of the pigs on the enhanced diet was decreased.
• The results have implications for type two diabetes risk factors in humans.

## 1 Introduction

Dietary fibre is an important component in a healthy diet. However, current knowledge does not explain all the physiological benefits associated with dietary fibre consumption. In the UK most people do not consume the recommended average intake for adults of 18 g (NSP) per day (Scientific Advisory Committee on Nutrition, 2015); the average intake is 12.8 g/day for women and 14.8 g/day for men. This is important because of its association with lowering for cardiovascular disease (CVD). The rate of obesity is increasing in the developed world resulting in increases in morbidity and mortality from CVD and metabolic disorders such as type-2 diabetes. In 2013, it was estimated that more than 60% of adults in the UK were overweight including 25% obese. Diabetes alone costs the NHS £10b per year and is associated with 24,000 deaths, half of which are CVD related (Hex, Bartlett, Wright, Taylor, & Varley, 2012). Clinical and epidemiological studies have demonstrated that diet and lifestyle changes are essential parts of a multifaceted approach to prevent and/or limit disease progression.

The use of dietary fibre is one tool that can be used to lower risk factors for cardiovascular disease and type 2 diabetes mellitus (Anderson et al., 2009). Recently, the InterAct consortium, using 10.8 years of follow-up of 11,559 participants with type 2 diabetes, was able to conclude that the overall evidence indicated that the intake of total and cereal fibre is inversely related to the risk of type 2 diabetes (InterAct Consortium, 2015). In addition, a number of studies have shown efficacy for a range of different dietary fibres. For example the cereal dietary fibre (CDF) β-glucan has been shown to lower cholesterol and EFSA allows a health claim on this basis (EFSA-NDA, 2011). Although the precise mechanism is not known, it is thought to involve the sequestering of bile acids, probably through entrapment of mixed micelles (Brownlee, 2011). Bile acids have also been shown to increase secretion of the satiety hormone GLP-1 as a result of binding to TGR5 in the distal ileum and colon, even when bound to a sequestrant (Prawitt, Caron, & Steels, 2014). Finally, bile acids have been shown to alter cholesterol homeostasis and lipid metabolism through binding to the farnesoid X nuclear receptor (FXR), both in the GI tract and in the liver.

The ability of dietary fibres to alter digestion and metabolism depends upon their physical properties and there are clearly large differences between soluble and insoluble fibre. However, these are normally consumed together for instance in porridge or bakery products. The initial effects in the upper GI tract are thought to be a consequence of the increase in viscosity that can be induced by soluble fibre (Kristensen & Jensen, 2011). The viscosity that can be imparted by soluble fibre depends on a number of factors including the molecular weight and the extent of hydration. In general, higher viscosity meals tend to cause increased gastric retention in comparison to the equivalent lower viscosity meal (Juvonen et al., 2009). In a recent *in vitro* study, the impact on digestion of the addition of oat bran to biscuits was assessed. In particular, the viscosity of the chime was measured throughout digestion (Villemejane, Wahl, Aymard, Denis, & Michon, 2015), showing that the viscosity was maintained throughout the gut up to the ileal compartment and that the highest viscosity was obtained with biscuits containing soluble fibre. The effect of viscosity on digestion was also determined and protein hydrolysis decreased with fibre enrichment, i.e. increased viscosity (Villemejane et al., 2016).

In addition to viscosity effects in the intestinal lumen and the consequent impact on gastrointestinal motility and rates of hydrolysis, the soluble fibre may interact with the intestinal mucus and decrease its permeability (Mackie, Macierzanka, Aarak, Ridout, & Bajka, 2015) or secretion rate. Previous studies in rats have shown that low-methoxyl pectin did not affect the number of goblet cells but could interact directly with the epithelium and stimulate small intestinal mucin secretion (Hino et al., 2013), although no explanation of the mechanism was provided. Two studies undertaken in pigs showed that the addition of cereal dietary fibre to a standard diet increased the flow and presumably the secretion of intestinal mucin (Morel et al, 2005, Morel et al, 2003). More specifically, the addition of β-glucan to a diet containing cellulose increased both mucin secretion and endogenous amino acid and nitrogen losses in the small intestine. The explanation given in these and other articles is that the dietary fibre increased the abrasion of the mucus layer (Montagne, Piel, & Lalles, 2004), presumably as a result the increase in luminal viscosity. Although it is unclear from these articles whether the secretion of mucin is upregulated it seems highly likely. The same trend has also been observed in rats fed on a range of dietary fibres (Brownlee, Havler, Dettmar, Allen, & Pearson, 2003), where the thickness of both the tightly and loosely adherent colonic mucus layers were found to increase as a result of the fibre in the diet.

In the work reported in this article we have used two different diets containing different amounts of oat meal as the fibre in order to assess the effect on the rheological properties and permeability of the intestinal mucus and mucin gene transcription.

## 2 Materials and methods

### 2.1 Animals and diets

Ten pigs in two groups of five raised at Easton Otley College were fed *ad libitum,* a standard commercial pig finisher diet (Easey Pigs, Eye, UK) either with or without supplementation with 10% oat bran (Suma Wholefoods, Elland, UK) for 3 days. The five OM10 fed animals were ear-tagged to allow carcass identification at the abattoir. Following feeding, the animals were transported to a local abattoir (Cranswick Country foods, Watton, Norfolk, UK) for conventional slaughter. Immediately following slaughter, porcine intestines were obtained and samples removed as outlined below.

The macronutrient composition of the two diets is described in Table 1. Water was also available to all of the animals throughout the three days. The control diet contained 0.7% cereal β-glucan, while the oat bran contained 8.7% oat β-glucan, giving a final value of 1.5% in the OM10 diet. The nutritional composition of the oat bran was 9.4% fat, 13.4% protein and 47.3% carbohydrate including 18.2% fibre. The average molecular weight of the β-glucan extracted from the oat bran by *in vitro* digestion and determined by HPLC-SEC was 2.3 MDa, subsequent treatment of the sample with laminarinase, completely abolished the peak, confirming it to be the β-glucan. The SEC used dextran Mw standards at 0.1, 0.18, 0.35 and 0.85 MDa. The differences between the conformation of the dextran and β-glucan may lead to a slight overestimation of the molecular weight of the β-glucan.

### 2.2 Sample collection

Porcine mucus was prepared as described previously (Macierzanka et al., 2011). Briefly, the fresh porcine small intestine obtained as described above, was stored on crushed ice for transport to the laboratory. The gut was rinsed through with ice cold phosphate buffer (10 mM phosphate pH 6.5, 5 mM EDTA) followed by a further rinse with the same buffer containing a protease inhibitor (0.5 mM Pefabloc, AEBSF). The gut was then opened out flat and mucus was collected by gently scraping the jejunal surface. Porcine jejunal mucus was isolated less than 45 min after slaughter. Samples were frozen in liquid nitrogen and stored at −80 °C for further use. Proximal small intestinal mucosal tissue samples were collected into RNALater (Sigma, Poole, UK), frozen in liquid nitrogen and stored at −80 °C for gene expression analysis.

### 2.3 Sample analysis

The β-glucan content of samples of the control (Control) and 10% oat bran (OM10) diets was analysed using a β-glucan mixed linkage kit (Megazyme, Co. Wicklow, Ireland). The β-glucan content of the soluble fractions of the digesta was determined using the method of Schmitt and Wise (2009). After brief centrifugation (3000 × g, 5 min, 1 °C) to pellet large fragments of insoluble material a portion of the supernatant (0.5 mL) was re-centrifuged (16,000 × g, 30 min, 1 °C) to remove fine particles of sediment, the resulting supernatant was diluted with four volumes of phosphate buffered saline solution (PBS) comprising 0.01 M phosphate buffer, 0.0027 M potassium chloride and 0.137 M sodium chloride at pH 7.4. Calcafluor (F3543, Sigma-Aldrich Company Ltd, Dorset, UK) was prepared in a 50 mM solution of sodium tetraborate as the Calcafluor was found to be almost insoluble in pure water and the Triton X100 was omitted as it had no practical effect. Additions of both internal standard (β-glucan) and Calcafluor were required to correct for quenching of both the sample and reagent fluorescence caused by components of the duodenal digesta. Fluorescence was measured on a FLUOstar Optima plate reader, Excitation 340 nm, Emission 420 nm (BMG Labtech Buckinghamshire, UK). 90% pure β-glucan from oats used as standard was kindly provided by the Swedish Oat Fibre company.

The starch content of samples of the two diets was measured using the Total Starch Assay Kit (AA/AMG), (Megazyme). The starch content of samples of digesta was performed on the insoluble residue of the sample after centrifugation (3000 × g, 20 min, 0 °C). Analyses of the samples of diet or digesta were both performed using the kit manufacturer's protocol for samples that may contain glucose and/or maltodextrins.

Soluble amine analysis was performed using o-phthaldialdehyde (OPA, Sigma) on samples of digesta after centrifugation (16,000 × g, 10 min, 0 °C) using the method of Cornec et al. (1998).

### 2.4 Bile acids

The concentration of total bile acids in intestinal mucus samples was performed using the bile acids (enzymatic cycling) test kit (Alpha laboratories, Hampshire, UK) after centrifugation (16,000 × g, 5 min, 1 °C) and analysed as per the kit manufacturer's protocol except that the volumes of sample and reagents were reduced to perform the assay in a micro titre plate format. Mucous was mixed with four volumes of PBS then centrifuged (16,000 × g, 30 min, 1 °C). The absorbance was measured at 405 nm using a Bio-Rad Benchmark Plus microtitre plate spectrometer (Bio-Rad, Hertfordshire, UK) operated in the kinetic mode.

The composition of bile found in the intestinal mucus samples was performed using an Agilent 1260 binary HPLC coupled to an AB Sciex 4000 QTrap triple quadrupole mass spectrometer (Sciex, Cheshire UK). HPLC used a binary gradient of solvent A (water + 5 mM ammonium acetate + 0.012% formic acid) and solvent B (methanol + 5 mM ammonium acetate + 0.012% formic acid) at a constant flow rate of 600 µL/min using a Supelco Ascentis Express C18 150 × 4.6, 2.7 µm column (Supelco, Dorset UK) maintained at 40 °C. The injection (5 µL) was made at 50% B, held for 2 min, ramped to 95% B over 20 min and held until 24 min before re-equilibration to starting conditions. Perdeuterated standards (D4) and unlabelled bile acid standards were obtained from Steraloids Inc. (Rhode Island, USA). The mass spectrometer was operated in the negative electrospray mode with capillary voltage of −4500 V at 550 °C. Instrument specific gas flow rates were 25 mL/min curtain gas, GS1: 40 mL/min and GS2: 50 mL/min. Quantification was performed using Analyst 1.6.2 software (Sciex, Cheshire UK) to integrate detected peak areas relative to the deuterated internal standards. Deuterated standards dissolved in 70% v/v methanol:water were added to the sample of mucous (250 µL) then the bile acids were recovered from the mucous using solid phase extraction on C18, 500 mg Bond Elute cartridges (Agilent, Cheshire, UK) using the method of Rod, Converse, & Hofmann (1987).

### 2.5 *In vitro* digestion of the diet

Two meals were used in the work described below. The first was the diet fed to the pigs and the second was a standardised emulsion system that we use to provide lipid digestion products. The pig diets were mixed thoroughly to avoid stratification then a 7 g sample was digested. The diets and the emulsion were digested using the standardised *in vitro* digestion protocol recommended by the Infogest COST Action (Minekus et al., 2014) involving 2 min oral digestion, then 120 min gastric digestion followed by 120 min of small intestinal digestion. Emulsions comprising 3.0 mg/mL sodium caseinate solution in 150 mm NaCl at pH 6.5 stabilising 18% triglyceride were prepared by passing a premix of oil and sodium caseinate for a total of 6 times at 20,000 psi through an LV1 Microfluidiser (Microfluidics, Massachusetts, USA).

### 2.6 Micro-viscosity

Particle tracking was carried out using carboxylated fluorescent latex beads with diameters of 500 nm (Sigma, Poole, UK) and 100 nm (Magsphere Inc, Pasadena, CA, USA) at 37 °C using a method modified from Macierzanka et al. (2011). Briefly, the latex beads were diluted 1 in 20 to a concentration of 0.125% w/v in Bis-Tris (0.15 M NaCl, 25 mM Bis-Tris) containing 7.4 mM Sodium Taurocholate and Sodium Glycodeoxycholate (Sigma, Poole, UK) at pH 6.5 to mimic human bile. The latex beads were added to the mucus (49 µL mucus: 1 µL latex bead mixture). Samples were gently mixed and loaded onto glass slides using 9 mm × 120 µm SecureSeal spacers (Sigma, Poole, UK). A Leica SP5 (II) laser scanning confocal microscope was used to image the motion of 500 nm beads at 40× magnification. At least 100 particles were tracked per sample for 200 frames at 500×ms/frame. A focal depth in the centre of the well was chosen to avoid interaction of the beads with the glass slide or coverslip. The motion of 100 nm latex beads was followed using a Leica SP5 (II) with an 8 kHz resonance scanner, ×63 objective lens and an imaging rate of 37×ms per frame. In both cases only particles that could be continuously tracked for a minimum of 50 frames were analysed. 2D bead trajectories, as projections of 3D motion were analysed, using Image-Pro Plus 7.0 software, to calculate time-dependant mean square displacement of particles (MSD) = Δr^2^(Δt) where r represents the movement of particles in the x and y directions and t represents time. Diffusion coefficients were calculated from the slope of the time-dependant MSD (D = MSD/4Δt).

### 2.7 Lipid diffusion

The ability of lipid from the digested diets and a model emulsion to diffuse through mucus were assessed by fluorescence imaging. Sample digesta was taken from the beginning and end of the simulated duodenal phase, 5 min and 120 min respectively. The lipid in the digested samples was visualised using Nile Red at a final concentration of 50 µM (Sigma, Poole, UK). Diffusion of lipid in mucus was assessed using fluorescence recovery after photo-bleaching (FRAP). Briefly, the stained digesta was mixed into mucus; samples were gently mixed and loaded onto glass slides using 9 mm × 120 µm SecureSeal spacers (Sigma, Poole, UK). The FRAP was conducted using a Leica SP5 (II) Laser scanning confocal microscope with an 8 kHz resonance scanner. A bleach spot of 50 µm diameter was used with an initial post bleach time of 3.7 s at 37×ms/frame, followed by 25 s at 250×ms/frame. Diffusion coefficients were calculated from the fluorescence recovery data using nonlinear least-square fitting as described by Ladha et al. (1996) using the following equation:

$$F\left(t\right)=\frac{F\left(0\right)+F\left(\infty \right)\left(t/\beta \tau D\right)}{1+\left(t/\beta \tau D\right)}$$

where F(t) is the observed fluorescence as a function of time, F(0) is the fluorescence immediately after bleaching, F(∞) is the fluorescence at infinite time after bleaching, β is the depth of bleach and τ_D_ is the diffusion time. The lateral diffusion coefficient, D is then given by D = ω^2^/4τ_D_, where ω is the radius of the bleach region.

### 2.8 Gene expression

RNA was extracted from homogenised mucosal tissue samples using an RNeasy mini kit (Qiagen, UK), treated with the DNase I (Sigma, UK), and reverse transcribed with Precision RT (Primer Designs, Southampton, UK) following the manufacturer's instructions. Quantitative real-time polymerase chain reaction (qRT-PCR) was performed with pig specific primers for *MUC1, MUC2, MUC4, MUC5ac* (Sigma Genosys, UK) designed based on sequences (including reference sequences) of the target genes (Table 2) using Primer-BLAST (https://www.ncbi.nlm.nih.gov/tools/primer-blast/). Real-time PCR was performed using a PrecisionPLUS SYBR Green PCR master mix (Primer Designs, Southampton, UK) and StepOne Real-Time PCR system (Applied Biosystems, Life Technologies). cDNA samples were assayed in duplicate, and gene expression levels for each sample were normalised (root mean square) relative to *GAPDH, GSR and GPI* reference genes (Primer Designs, Southampton, UK) with 2^−ΔΔCt^ calculation.

### 2.9 Statistics

All data are represented as the mean ± sem (standard error of the mean) unless otherwise stated. Diet macronutrient content, bile salt percentages and diffusion of digested diet through mucus were compared by 2-way analysis of variance (ANOVA) with Bonferroni multiple comparison post-hoc analysis. Diffusion coefficient data generated by multiple particle tracking and comparisons of individual bile salts were compared using an un-paired, one-tailed t-test with a 95% confidence interval.

## 3 Results

In order to assess the differences that might be seen *in vivo***,** the two diets used in the study were digested *in vitro* using the Infogest protocol as described above. Analysis of the β-glucan content of the two diets yielded values of 0.7% and 1.4% for the Control and OM10 diets respectively. However, subsequent analysis of the soluble component in the simulated gastric phase gave values of 0.26 ± 0.03 mg/mL and 0.39 ± 0.08 mg/mL for the Control and OM10 diets respectively and in the duodenal phase the concentrations were 0.66 ± 0.32 mg/mL and 1.62 ± 1.32 mg/mL for the Control and OM10 diets respectively. These comparatively low levels are partly due to the dilution effects of the digestion protocol. However, following correction for dilution, only 15% and 11% of the available β-glucan was released from the Control and OM10 meals respectively during the gastric phase. This is in marked contrast to the simulated duodenal phase where there is a further dilution by a factor of two so the proportional release was 75% and 92% from the Control and OM10 respectively.

**Fig. 1:**
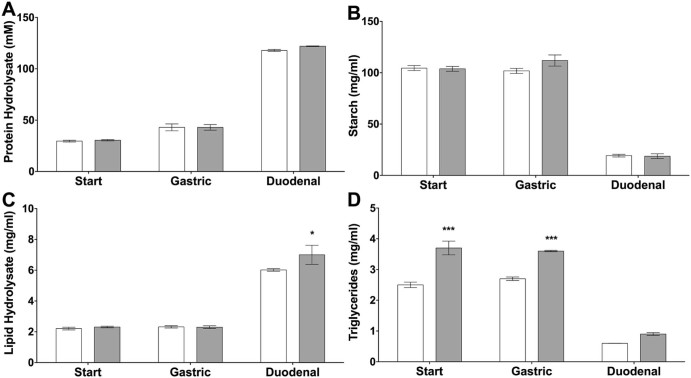
Analysis of the macronutrient content of the Control and OM10 diets. Protein hydrolysis is shown in A, starch content in B, lipid hydrolysis products (mono- and di-glycerides and free fatty acids) in C and remaining triglycerides are shown in D. Data represented as the mean ± sem, n = 5 per group. Statistical significance was determined using a 2-way ANOVA (*P < 0.5 and ***P < 0.001 vs control Diet).

The extent of protein hydrolysis during the digestion was followed using the OPA method as described above. Fig. 1A shows limited proteolysis in the gastric phase and significantly more hydrolysis in the duodenal phase. There was no significant difference in the extent of protein hydrolysis between the two diets. The amount of starch remaining in the digesta from the gastric phase was the same as in the starting meal and this is expected given the protocol used. However, more than 80% of the starch was hydrolysed in the duodenal phase of the simulation as shown in Fig. 1B. Fig. 1C and D shows that the addition of 10% oat bran not only increased the β-glucan content but also added significantly to the triglyceride load of the diet as the oat bran contained 8% lipid. The addition of the oat bran to the control diet had no effect on the hydrolysis of lipid. However, given the low concentration of β-glucan in the duodenal phase of the digestion, this is perhaps not surprising.

One of the main health related effects of BG is the sequestering of bile and its potential to alter bile secretion. In order to investigate this property the concentration of bile acid was measured in the mucus from the proximal small intestine of the pigs after consumption of both diets. The concentrations were 1.47 ± 1.4 and 1.51 ± 1.2 mM for the Control and OM10 diets respectively. A detailed analysis of the bile salts in intestinal mucus samples from the two groups of pigs is given in Table 3.

**Fig. 2:**
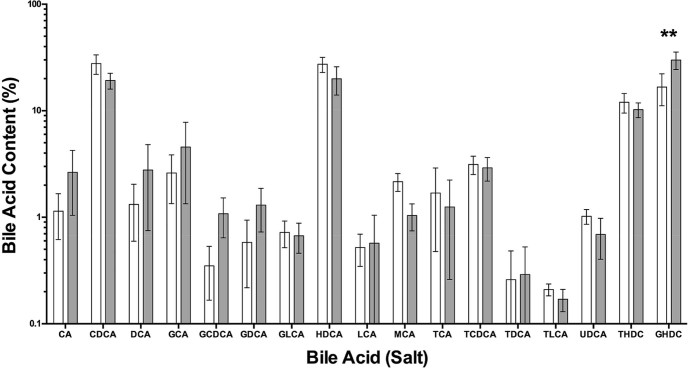
The average bile acid composition (% of total bile) in mucus samples taken from the intestines of pigs fed either the control diet (white) or OM10 diet (grey) plotted on a log scale. Data are presented as the mean ± sem, n = 5 per group. Statistical significance was determined using a 2-way ANOVA (**P < 0.01 vs control Diet).

The results are also summarised in Fig. 2 and show that there were significant differences in the proportion of only three different bile acids/bile salts between the two groups. Individual t-tests demonstrated an increase in GCDCA and GHDC but a decrease in MCA (P < 0.05). In total, significant concentrations of 19 different bile acids were measured. Although there was a broad spread of bile compounds present, over 82% of the bile was accounted for by just 4 compounds, namely CDCA and HDCA and its two conjugated bile salts, THDC and GHDC. Overall significance determined by 2-way ANOVA showed no effect of diet (P = 0.99), however GHDC was significantly increased in pigs fed the OH10 diet.

**Fig. 3:**
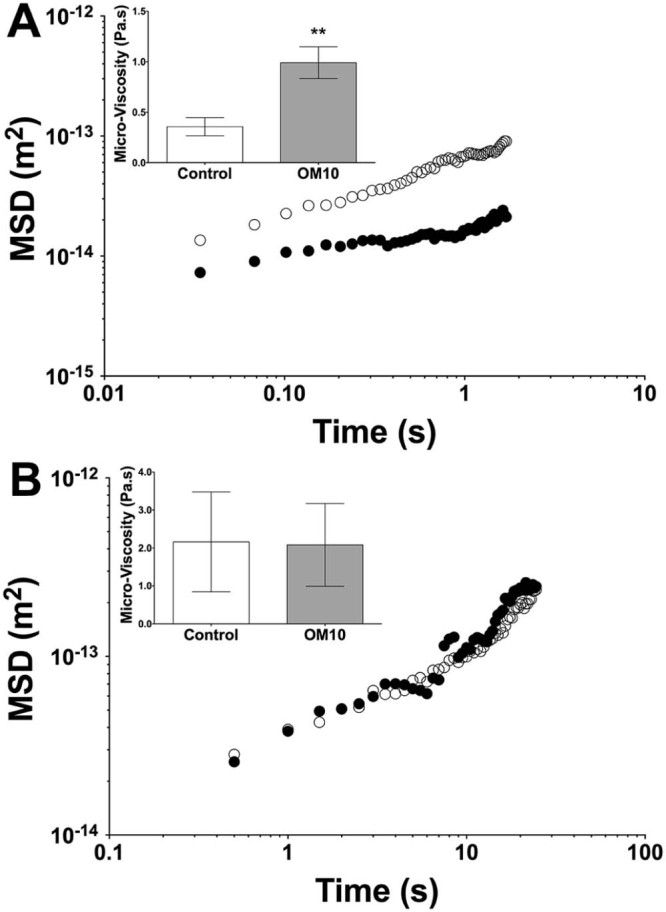
Assessment of the mobility of (A) 100 nm and (B) 500 nm bile salt coated carboxylated polystyrene beads through small intestinal mucus ex vivo from pigs fed Control (open circles) or OM10 diets (closed circles). Mean squared displacement values were calculated over 50 frames (1.85 s for 100 nm beads and 25 s for 500 nm beads) and used to derive a Stokes viscosity (inserts). The viscosity is presented as the mean ± sem, n = 5. Statistical significance was determined using Students t-test (**P < 0.01 vs control Diet).

The impact of the OM10 diet on the properties of intestinal mucus was determined in a number of different experiments. Firstly the permeability of the mucus to both 100 nm and 500 nm carboxylated latex beads was measured by particle tracking. The ensemble mean square displacement (<MSD>) plotted as a function of time is shown in Fig. 3 for both diameters of bead. Fig. 3A clearly shows that the 100 nm beads were able to diffuse more quickly through the intestinal mucus from the pigs on the Control diet than the OM10 diet. The mean diffusion coefficients were calculated from the MSDs and the Stokes–Einstein equation used to calculate the Stokes viscosity. The difference in MSD is also reflected in the significant difference in Stokes viscosity between the two diets with the OM10 diet increasing the apparent viscosity by a factor of two. This is in contrast to the data for the 500 nm latex beads in Fig. 3B, which showed no difference in diffusion between the two diets. The value of the Stokes viscosity calculated from the data for 500 nm beads was more than 4 times higher for the control diet, suggesting that what has really been shown is the difference in permeability between the mucus samples resulting from the two diets. The calculated viscosities are shown in the insets to Fig. 3.

**Fig. 4:**
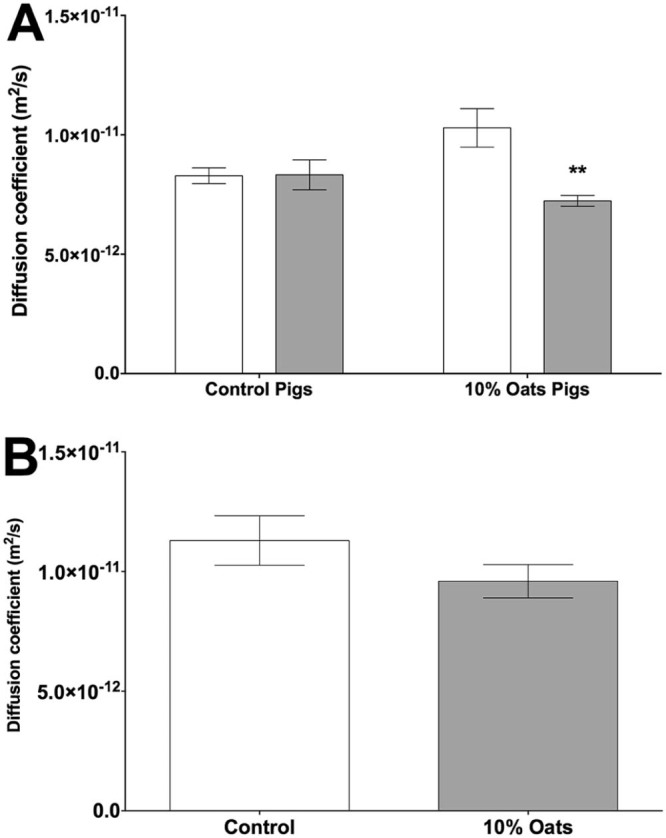
The diffusion coefficient of fluorescently labelled lipid from (A) the pig diet and (B) an emulsion following 120 min simulated duodenal digestion in intestinal mucus from pigs fed control or OM10 diets. The data are presented as the mean diffusion coefficient of lipid from the pig diets (open and closed bars represent digested control or OM10 diets respectively). The values shown are the mean ± sem, n = 5. Statistical significance was determined using a 2-way ANOVA (**P < 0.01 vs control Diet).

In addition to demonstrating that the OM10 diet decreased the permeability of the intestinal mucus to 100 nm latex beads, we also aimed to show an effect on the diffusion of the digested diet itself. In order to provide consistent material, both the Control and OM10 diets were digested *in vitro* as described above and in addition we used digesta from a standard protein stabilised emulsion as described previously (Mackie et al., 2015). The lipid in the digesta was then fluorescently labelled with Nile Red and the diffusion coefficient was determined by FRAP. The mean diffusion coefficients of digesta from both diets were the same in intestinal mucus from pigs fed on the Control diet, as shown in Fig. 4. However, in the mucus from the pigs fed the OM10 diet the mean diffusion coefficients of the digesta from the Control and OM10 diets showed significant differences. The OM10 digesta gave a lower mean diffusion coefficient than the Control digesta. The mean diffusion coefficient of the digesta from the standard emulsion was also slower in the mucus from the OM10 diet pigs than the mucus from the Control diet pigs although the difference was not statistically significant.

In addition to measuring the biophysical properties of the intestinal mucus we also assessed whether there were any differences in mucin gene expression. The normalised data from the qRT-PCR are shown in Table 4 as mean relative abundance and standard deviation. The genes shown are the primary genes associated with the secreted (MUC2 and MUC5ac) and membrane bound (MUC1 and MUC4) mucins found in the small intestine. No differences in gene expression were found between the pigs fed on the two diets.

## 4 Discussion

The dietary fibre β-glucan has been shown to have an effect on cholesterol homeostasis. This is thought to be related to increased excretion of bile acids (Brownlee, 2011) and the subsequent conversion of cholesterol to bile in the liver.

In this study we increased the β-glucan content of the diet of five healthy pigs from 0.7% to 1.5% for a period of three days. The simulated digestion of the two diets in Fig. 1 showed that this increase was not sufficient to impact macronutrient digestion and this is probably because, even though the soluble β-glucan level in the simulated intestinal phase was over 90% of the maximum possible based on the initial concentration in the food, the concentration was only 0.16% in the OM10 diet and the increase in viscosity from such a concentration would have been minimal (Agbenorhevi, Kontogiorgos, Kirby, Morris, & Tosh, 2011).

The action of dietary fibre has been shown to have strong implications for circulating bile (Ellegard, Andersson, 2007, O'Keefe et al, 2015). In particular, it can limit bile reabsorption and draw more bile into the colon where it can be modified by bacterial enzymes (Ridlon et al, 2013, Ridlon et al, 2014). Although porcine bile includes a number of different bile salts, they can be simplified into essentially two groups (Qiao et al., 2011). These are hyodeoxycholic acid (HDCA) and chenodeoxycholic acid (CDCA), which make up over 90% of porcine bile secreted into the intestine. These two bile acids differ only in the addition of a hydroxyl group in the 6α position in HDCA. In addition, the bile acids are conjugated with either glycine or taurine and in porcine bile the glycine form is predominant. The primary bile acid CDCA undergoes 7α-dehydroxylation by a limited range of intestinal anaerobic bacteria (Ridlon, Kang, & Hylemon, 2006) to lithocholic acid. However, bile acids must be deconjugated by bile salt hydrolase before 7α-dehydroxylation occurs. In this study we recovered samples of mucin from the proximal small intestine of pigs, in which we were able to measure the bile composition. The standard deviations in Table 3 and for the total bile concentrations indicate that the amount of bile in the different animals varied significantly, as one might expect from animals with an *ad libitum* feeding regime. Despite the large variation in bile concentrations, the mean composition was similar between the two groups of animals. However, there was one component that showed a significant difference. There was an increase in the conjugated secondary bile salt GHDC that is formed as a result of conjugation of HDCA, which is in turn formed from DCA by bacterial enzymes. It could be argued that an increase in GHDC suggests that more bile was being delivered to the colon before being reabsorbed as a result of consuming the OM10 diet. The bile acid HDCA, when administered to mice, has been shown to exert a hypolipidaemic effect in mice, in which downregulations of de novo lipogenesis and desaturation of saturated fatty acids are suggested to play important roles (Watanabe & Fujita, 2014). However, the increase in HDCA seen in this study is in contrast to a recent study in pigs where a diet enriched in wheat arabinoxylan fed for four weeks led to a significant decrease in HDCA in the gall bladder (Gunness et al., 2016).

The soluble dietary fibre sodium alginate has previously been shown to decrease the permeability of intestinal mucus (Mackie et al., 2015). Thus it might be expected that similar behaviour might be seen with the soluble fibre fraction in the intestinal mucus from the pigs. Although the difference in soluble β-glucan concentrations from the two diets *in vitro* was only 0.1%, this was still sufficient to decrease the permeability of the intestinal mucus to 100 nm beads *ex vivo*, as shown in Fig. 3A. This demonstrates a decrease in the pore size of the mucin network at the 100 nm scale. The micro-viscosity calculated from the motion of the 100 nm latex beads was 1 Pas for the OM10 diet, which was more than twice the value for the mucus from the Control diet. The data for the 500 nm latex beads showed Stokes viscosity values higher still at 2 Pas for both diets. This is to be expected as the beads were clearly above the mean network size for the mucus from pigs fed on both diets. A range of studies have previously shown that the mucin network pore size is around 100 nm in mucus from a range of sources including cervical and intestinal (Bajka et al, 2015, Olmsted et al, 2001).

Of course the intention of the work described here was not to study the diffusion of latex beads but of nutrients from digesta. In particular, the low aqueous solubility of lipids means that the products of lipid hydrolysis are transported as self-assembled mixed micelles and as such were the main target of this investigation. Fluorescently labelled digesta samples from the end of simulated digestion were used to probe the permeability of the mucus as shown in Fig. 4. The mean diffusion coefficient is a function of the mean size of the diffusing particles and the fact that both diets gave the same value in the mucus from the pigs fed on the control diet suggests that they had a similar mean size. However, in the intestinal mucus collected from the pigs fed on the OM10 diet, the diffusion of the Control digesta was faster than the diffusion of the digesta from the OM10 diet. Thus the combination of feeding the pigs on an OM10 diet for three days and adding the extra fibre in the OM10 diet decreased the permeability of the mucus to the digesta. A similar trend, although not statistically significant, was seen with the digesta from a standard protein stabilised emulsion.

While the ability of dietary fibre to decrease rates of nutrient absorption and therefore lower glycaemic response is not new (Brennan, 2005, Malkki, Virtanen, 2001, Scazzina et al, 2013), the evidence for prolonging postprandial triglyceride absorption remains inconsistent (Goff et al, 2013, Tiihonen et al, 2015). However, this is the first time that it has been shown, at least in part to be due to an interaction with the intestinal mucus layer. The data in Fig. 3, Fig. 4 have already shown that the increased fibre in the OM10 diet was able to decrease the permeability of intestinal mucus. In addition, it has previously been shown that increasing dietary fibre can increase mucus production (Hino et al, 2013, Paturi et al, 2013). Therefore, we also analysed samples of small intestinal tissue in order to determine whether the genes associated with mucus secretion were upregulated. Neither the primary glycocalyx mucin MUC1 nor the primary secreted mucin MUC2 showed any significant increase in gene expression following feeding of the OM10 diet for 3 days (Table 4). However, as changes in transcription are likely to be an adaptation to changes in the environment of the lumen or mucus layer rather than a direct effect between fibre and the epithelium, a longer feeding period may be required. Previous work in pigs has suggested that physiological responses such as bile acid synthesis and mucosal gene expression are not only dependant on dietary composition but also on adaptation (Gunness et al., 2016). Similarly, MUC4 and MUC5ac were not significantly altered (Table 4), although their level of expression was very low (data not shown).

Other components in cereal dietary fibre that have evoked significant interest are the phenolic compounds. These have been mooted as possible cross-linking agents that may alter the permeability mucus. However, in previous work we were able to show that this is not the case for intestinal mucus (Gonzales et al., 2015). Indeed the kaempferols showed no interaction with intestinal mucus. In addition, it has been shown that the phenolic compounds from cereals generally have low bioavailability as they are trapped in the insoluble polysaccharides of the bran layer (Wang, He, & Chen, 2014).

## 5 Conclusions

This article describes a preliminary study investigating the effect of doubling the β-glucan content of a porcine diet for 3 days and focussed on the properties of the intestinal mucus layer. *In vitro* digestion of the OM10 and control meals showed that over 90% of the β-glucan was released from the OM10 diet in the simulated proximal small intestine, although this did not alter rates of nutrient hydrolysis. Measurements of the permeability of the porcine intestinal mucus showed that the OM10 diet decreased permeability to 100 nm latex beads and more importantly reduced permeability to lipid from the digested OM10 diet. The dietary fibre, β-glucan, has been shown to lower cholesterol by reducing bile recycling. The data shown here suggest for the first time that reducing mass transfer of bile and lipid through the intestinal mucus layer may be one way in which this decrease in bile reabsorption is enabled and that postprandial lipid absorption is prolonged.

## Figures and Tables

**Table 1:** The macronutrient of the control (55–100 finisher diet) and enhanced β-glucan diet composition (%).

	Control	OM10 (10% oat bran)
Crude protein	16.59	16.03
Crude fat	4.00	4.40
Carbohydrate	74.75	73.28
Fibre (NDF)	15.92	23.33
β-Glucan	0.7	1.4
Gross energy (MJ/kg DM)	13.35	13.52

**Table 2:** A list of the qPCR primers used to follow expression of intestinal mucin genes.

Gene name	Accession number	Primers	T_m_ (°C)
MUC1	XM_001926883.1	F: TTAAGTGCTGTGAGCGCAAC	59.5
		R: TCTGGTAGGGCTGATGGTCA	60.0
MUC2	AK231524.1	F: AGGAAATGCATCCCTCGCAA	60.0
		R: GGAGCAGTCGTTCATGGTCA	60.0
MUC4	XM_005670136.2	F: TTTCTGGAGCCATGAGGGGG	61.6
		R: GTCATAGTGTTTCCACCCAGGAC	60.9
MUC5ac	AF054584.1	F: CGTAGAGCACAGGTGCAAGT	59.0
		R: GCAGGGTCACGTTTCTCAG	59.0

**Table 3:** The concentrations (µM) of the bile acids found in intestinal mucus samples. The data are given as the mean and standard deviation.

Bile acid	OM10		Control	
	Mean	SD	Mean	SD
α-Muricholic acid (α-MCA)	1.58	1.53	0.91	0.46
β-Muricholic acid (β-MCA)	0.53	0.44	1.07	0.96
Cholic acid (CA)	29.25	37.56	6.14	5.24
Chenodeoxycholic acid (CDCA)	290.12	241.84	430.34	392.05
Deoxycholic acid (DCA)	25.97	34.26	6.37	2.37
Glycocholic acid (GCA)	31.65	20.67	14.03	13.79
Glycochenodeoxycholic acid (GCDCA)	23.97	34.84	2.37	1.95
Glycodeoxycholic acid (GDCA)	28.42	41.75	2.85	2.12
Glycolithocholic acid (GLCA)	16.86	19.33	16.91	19.93
Hyodeoxycholic acid (HDCA)	258.74	190.78	351.92	304.51
Lithocholic acid (LCA)	3.05	2.62	6.20	6.23
Muricholic acid (MCA)	15.89	20.43	52.04	49.48
Tauro-α-mirocholic acid (T-α-MCA)	0.00	0.00	0.25	0.39
Taurocholic acid (TCA)	12.41	11.10	4.73	2.46
Taurochenodeoxycholic acid (TCDCA)	63.34	59.48	66.93	71.43
Taurodeoxycholic acid (TDCA)	2.94	3.31	0.94	0.40
Taurolithocholic acid (TLCA)	4.13	5.08	5.45	6.38
Ursodeoxycholic acid (UDCA)	8.60	10.87	22.32	25.81
ω-Muricholic acid (ω-MCA)	1.96	1.95	4.06	3.93
Taurohyodeoxycholate (THDC)	214.52	205.29	249.05	266.52
Glycohyodeoxycholate (GHDC)	472.94	330.72	228.90	299.93

**Table 4:** Relative abundance (2^−ΔΔCt^) of mucin genes in proximal small intestinal mucosa of pigs fed control or 10% oat bran diets. ΔΔCt was calculated using the average of 3 housekeeping genes (*GAPDH, GSR and GPI)*. The data are given as the mean and standard deviation, n = 5.

Gene name	Relative abundance		SD	Significance (P value)
MUC1	Control	1.000	0.573	0.375
	OM10	1.196	0.926	
MUC2	Control	1.000	0.676	0.431
	OM10	0.917	0.442	
MUC4	Control	1.000	0.696	0.279
	OM10	0.377	0.386	
MUC5ac	Control	1.000	0.776	0.140
	OM10	0.347	0.341	
